# Pregnancy- and Abortion-Related Mortality in the US, 2018-2021

**DOI:** 10.1001/jamanetworkopen.2025.54793

**Published:** 2026-01-21

**Authors:** Maria W. Steenland, Kerra Mercon, Benjamin P. Brown, Marie E. Thoma

**Affiliations:** 1 Department of Family Science, School of Public Health, University of Maryland, College Park, College Park; 2 Department of Obstetrics and Gynecology, Women and Infants Hospital of Rhode Island, Alpert Medical School, Brown University, Providence

## Abstract

**Importance:**

The 2022 Supreme Court decision *Dobbs v. Jackson Women’s Health Organization* and the subsequent legal restrictions to abortion have prompted national discussion about the importance of abortion access for maternal health. A commonly cited statistic in these debates, based on data from 1998 to 2005, is that the risk of death associated with childbirth is approximately 14 times higher than that of abortion.

**Objective:**

To estimate the ratio of pregnancy-related to abortion-related mortality using updated data (2018-2021), capturing increased detection of maternal death and increased safety of abortion since the original estimate.

**Design, Setting, and Participants:**

This cross-sectional study included all births (live and stillbirths) and pregnancy-related deaths from the National Vital Statistics System from 2018 to 2021. The number of abortion related deaths was obtained from the Pregnancy Mortality Surveillance System, and the number of abortions was obtained from the Guttmacher Institute. Data were analyzed between February and October 2025.

**Main Outcomes and Measures:**

Pregnancy-related deaths were identified using *International Statistical Classification of Diseases and Related Health Problems, Tenth Revision* (*ICD-10*) codes A34, O10 to O95, O96, O98 to O99. Pregnancy- and abortion-related mortality ratios were calculated per 100 000 births and abortions, respectively, per year and overall. To calculate the ratio, the pregnancy-related mortality rate per 100 000 births was divided by the abortion-related mortality rate per 100 000 abortions. Sensitivity analyses excluded *ICD-10* codes commonly misclassified as pregnancy related and COVID-19–related deaths.

**Results:**

During the period between 2018 and 2021, there were 14 902 571 births and 3 662 580 abortions. The mean ratio between pregnancy-related and abortion-related mortality was 69.6 (range, 52.9-105.2). Excluding nonspecific causes of pregnancy-related death, the mean pregnancy- to abortion-related mortality ratio was 52.9 (range, 38.2-74.2). Further excluding COVID-19–related mortality, the ratio was 44.3 (range, 34.5 to 74.2).

**Conclusions and Relevance:**

This cross-sectional study found that the ratio of pregnancy- to abortion-related mortality from 2018 to 2021 ranged from 44.3 to 69.6, at least 3 times higher than the ratio of 14.7 calculated using data from 1998 to 2005. These findings suggest that by taking away the option to end a pregnancy, abortion bans force pregnant people to take on the increased health risks associated with continued pregnancy.

## Key Points

**Question:**

What is the ratio between pregnancy-related and abortion-related mortality?

**Findings:**

This cross-sectional study using national data on annual pregnancy-related and abortion-related deaths, the annual number of abortions (3 662 580 abortions), and the annual number of births (14 902 571 births) between 2018 and 2021 found that the ratio between pregnancy- and abortion-related mortality in the study period ranged from 44.3 to 69.6, at least 3 times higher than the ratio of 14.7 calculated using data from 1998 to 2005.

**Meaning:**

These findings suggest that by taking away the option to end a pregnancy, abortion bans force pregnant people to take on the substantially increased health risks associated with continued pregnancy.

## Teaser

This cross-sectional study estimates the ratio of pregnancy-related mortality to abortion-related mortality in the US from 2018 to 2021.

## Introduction

Since the 2022 Supreme Court decision *Dobbs v. Jackson Women’s Health Organization* overturned *Roe v. Wade*, 13 states implemented total abortion bans and an additional 6 states implemented bans limiting the gestational weeks when abortion is allowable to between 6 and 15 weeks.^1^ Abortion restrictions infringe on bodily autonomy and have important consequences for pregnancy-related care and maternal health more broadly. By taking away the option to end a pregnancy, abortion bans require pregnant people to take on the increased health risks associated with ongoing pregnancy. The *Dobbs* decision and the subsequent legal restrictions to abortion in the US have prompted national discussion about the importance of abortion access for maternal health. A commonly cited statistic in these debates is that the risk of death associated with childbirth is approximately 14 times higher than that of abortion.^2^ This statistic comes from a study by Raymond and Grimes^2^ published in 2012 using data from the Pregnancy Mortality Surveillance System (PMSS) collected between 1998 and 2005.^2^

Although the study by Raymond and Grimes^2^ is widely used in discussions of the potential harms of the post-*Dobbs* abortion policy environment,^3,4,5^ the statistic may not reflect the increased risk associated with pregnancy relative to abortion in a more current context. Several changes over the past 2 decades may have affected this ratio. First, the 2003 revision of the US death certificate, which was fully implemented in 2018, added a checkbox indicating pregnancy status in the year before death.^6^ The addition of the checkbox improved identification of previously underreported maternal deaths, leading to higher estimates of measured maternal mortality between 2000 and 2019.^7,8,9^ Independent from the pregnancy checkbox, the COVID-19 pandemic increased the risk of mortality and morbidity related to pregnancy and childbirth in 2020 and 2021.^10^ Additionally, the safety of abortion may have increased due to the increase in abortions taking place in very early pregnancy, when potential risks are least likely.^11^ Over the last several decades, the percentage of abortions before 7 weeks gestation increased from 23% in 1999 to 40% in 2022.^12,13^ In this study, we re-estimate the ratio of pregnancy-related to abortion-related mortality using updated data from 2018 though 2021, capturing the changes to maternal death detection and in the safety of abortion since the 2012 estimate.

## Methods

This cross-sectional study used only aggregated data sources without individual identifying information and was thus not considered human participant research per federal and University of Maryland definitions. This study is reported following the Strengthening the Reporting of Observational Studies in Epidemiology (STROBE) reporting guideline

### Data Sources to Measure US Pregnancy-Related Deaths

There are 2 national systems that release annual data on pregnancy-related deaths: the National Vital Statistics System (NVSS) and the Centers for Disease Control and Prevention (CDC) Pregnancy Mortality Surveillance System (PMSS). The NVSS, administered by the National Center for Health Statistics, is based on the compilation of state death certificate information. Pregnancy-related deaths are determined by *International Statistical Classification of Diseases and Related Health Problems, Tenth Revision* (*ICD-10*) cause-of-death codes and a checkbox indicating whether the death occurred during or within 1 year from the end of a pregnancy.^14^ PMSS uses the same death certificate information alongside other sources (eg, PMSS links death certificates to birth certificates or fetal death reports [when available], media reports, and reports from public health agencies) and further reviews to confirm the death was pregnancy related.^15,16^ Earlier PMSS reports provided a breakdown of pregnancy-related deaths by the outcome of pregnancy (eg, live birth, stillbirth, ectopic, undelivered, unknown).^17^ This made it possible for Raymond and Grimes^2^ to generate a ratio with deaths among persons with a live birth in the numerator and total live births in the denominator.^2^ However, the most recently released PMSS data are no longer disaggregated by pregnancy outcome nor are these data accessible to researchers for further disaggregation.^15^ Therefore, we used publicly available NVSS data (births, fetal deaths, and multiple cause-of-death data) accessed through CDC WONDER for this study and compared this to overall PMSS data available from CDC reports.^18,19^

### *ICD-10* Code Definition for Pregnancy-Related Mortality

Pregnancy-related mortality is defined as death while pregnant or within 1 year of pregnancy termination from a condition or event related to or aggravated by the pregnancy. We determined these deaths using *ICD-10* cause-of-death codes used to indicate maternal mortality (ie, death while pregnant or within 42 days of termination of pregnancy, A34, O00-O95, and O98-O99) and late maternal death (ie, within 43 days to 1 year of the termination of pregnancy, O96), consistent with other studies.^20^ Pregnancy-related mortality ratios were calculated by dividing pregnancy-related deaths between 2018 and 2021 by the number of births (live and stillbirths) for the corresponding years. This served as our starting point for analysis. We modified this classification of pregnancy-related mortality in 3 ways to account for timing of death during pregnancy, pregnancy misclassification, and the COVID-19 pandemic. Thus, providing a range of pregnancy-related mortality ratios for comparison under different assumptions.

First, we excluded *ICD-10* codes indicating a pregnancy-related death in the absence of a birth (ie, pregnancy with abortive [spontaneous or induced] outcome, *ICD-10* codes O00-O07). We did this to remove deaths among people who would not be captured in the denominator (ie, deaths earlier in pregnancy not resulting in a birth), upwardly biasing our estimate. Removing pregnancies with an abortive outcome (ie, molar and ectopic pregnancy, spontaneous and induced abortion) moves this study’s definition closer to Raymond and Grimes,^2^ who also excluded deaths among persons with these pregnancy outcomes. This served as our primary definition of pregnancy-related deaths: deaths during or within 1 year from the end of pregnancy and identified using *ICD-10* codes A34, O10 to O95, O96, and O98 to O99 indicating the underlying cause of death. Second, we excluded *ICD-10* codes with a higher likelihood of being misclassified as pregnancy related. Previous literature has demonstrated that the pregnancy checkbox used in NVSS data may result in false positives (ie, deaths of persons who were not pregnant).^21^ To account for the potential overcounting of maternal deaths, we conducted a second analysis removing nonspecific causes of pregnancy-related mortality (*ICD-10* codes O26.8, O95, and O99.8), which have been shown to have a higher likelihood of being misclassified as pregnancy related.^22,23^ Third, we excluded COVID-19–related deaths. The COVID-19 pandemic was associated an increase in pregnancy-related deaths of 49% at the peak in 2021.^20^ By 2022 and 2023, pregnancy-related mortality had declined to close to prepandemic levels.^24^ Therefore, we excluded deaths in which COVID-19 (*ICD-10* code U07.1) was listed as a multiple cause code to report a pregnancy-related mortality ratio that more closely reflects mortality outside of the height of the pandemic. Finally, we compared our modified estimates of the pregnancy-related mortality ratio using NVSS data with overall rates reported by CDC using PMSS data.

### Measurement of Abortion-Related Mortality

Abortion-related mortality was calculated according to prior convention. Abortion-related deaths were obtained from PMSS.^13^ PMSS defined an abortion-related death as “a death resulting from a direct complication of an abortion (legal or illegal), an indirect complication caused by a chain of events initiated by an abortion, or an aggravation of a pre-existing condition by the physiologic effects of abortion.” To identify abortion-related deaths, the CDC uses state or jurisdiction vital records, media reports, or individual case reports by public health agencies. For deaths that may be related to abortion, the CDC requests clinical records and autopsy reports.^13^ The number of abortions per year was obtained from the Guttmacher Institute,^25^ the most complete source for abortion surveillance data in the US.^13^ Abortion counts for 2021 were not available from the Guttmacher Institute. Therefore, consistent with the CDC’s 2022 abortion surveillance report,^13^ we assumed that the number of abortions in 2021 was the same as in 2020. Abortion-related mortality rates were calculated as the number of abortion-related deaths divided by the number of abortions, multiplied by 100 000.

### Statistical Analysis

To calculate the pregnancy- to abortion-related mortality ratio, we divided the pregnancy-related mortality rate per 100 000 live births by the abortion-related mortality rate per 100 000 abortions. Rates were calculated by year and overall, between 2018 and 2021. We selected 2018 to start this analysis because 2018 was the first year that the pregnancy checkbox was implemented in all states. The final year of the analysis is 2021 because this is the last year that the CDC released abortion-related mortality surveillance data. Data were analyzed between February and October 2025.

## Results

During the period between 2018 and 2021, there were 14 902 571 births and 3 662 580 abortions. The Figure presents a flowchart illustrating our *ICD-10* code definition for pregnancy-related death. Between 2018 and 2021 there were 4945 pregnancy-related deaths identified using the National Center for Health Statistics definition for maternal mortality in addition to late maternal deaths. After excluding 130 deaths among people with an abortive outcome, there remained 4815 pregnancy-related deaths during the study period. After removing 1153 nonspecific causes of pregnancy-related mortality (*ICD-10* codes O26.8, O95, and O99.8), there were 3662 deaths. Finally, there were 3065 pregnancy-related deaths during the study period after removing 597 COVID-19–related deaths.

**Figure.**
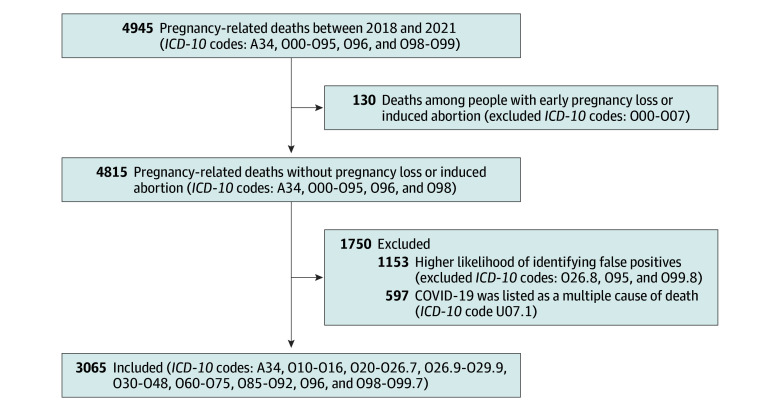
*ICD-10* Code Definitions of Pregnancy-Related Deaths Used in This Study *ICD-10* indicates *International Statistical Classification of Diseases and Related Health Problems, Tenth Revision*.

During the period between 2018 and 2021, the mean ratio of pregnancy-related to abortion-related mortality using our primary definition of pregnancy-related mortality was 69.6 (Table 1). The ratio ranged from 52.9 in 2020 to 105.2 in 2018 (Table 1). After excluding nonspecific causes of pregnancy-related death, the mean pregnancy- to abortion-related mortality ratio was 52.9 (range, 38.2-74.2) (Table 2). Further excluding COVID-19–related mortality, the ratio was 44.3 (range, 34.5-74.2) (Table 3). The pregnancy- to abortion-related mortality ratio calculated using PMSS data was 49.8 (range, 38.6-76.1) (Table 4).

**Table 1.** Ratio of Pregnancy-Related Mortality to Abortion-Related Mortality, 2018-2021^a^

Year	Pregnancy-related deaths, No.	Births (live births and still births), No.	Pregnancy-related mortality rate, No. per 100 000 births	Abortion-related deaths, No.	Abortions, No.	Abortion-related mortality rate, No. per 100 000 abortions	Ratio
2018	906	3 814 053	23.8	2	885 800	0.226	105.2
2019	1050	3 768 908	27.9	4	916 460	0.436	63.8
2020	1241	3 634 363	34.1	6	930 160	0.645	52.9
2021	1618	3 685 247	43.9	5	930 160	0.538	81.7
Total	4815	14 902 571	32.3	17	3 662 580	0.464	69.6

^a^ This estimate of pregnancy-related mortality is based on the following *International Statistical Classification of Diseases and Related Health Problems, Tenth Revision* codes: A34, O10-O95, O96, O98-O99. This excludes pregnancies with abortive outcomes (O00-O07).

**Table 2.** Ratio of Pregnancy-Related Mortality to Abortion-Related Mortality After Removing Nonspecific Causes of Pregnancy-Related Death, 2018-2021^a^

Year	Pregnancy-related deaths, No.	Births (live births and still births), No.	Pregnancy-related mortality rate, No. per 100 000 births	Abortion-related deaths, No.	Abortions, No.	Abortion-related mortality rate per 100 000 abortions, No.	Ratio
2018	639	3 814 053	16.8	2	885 800	0.226	74.2
2019	750	3 768 908	19.9	4	916 460	0.436	45.6
2020	896	3 634 363	24.7	6	930 160	0.645	38.2
2021	1377	3 685 247	37.4	5	930 160	0.538	69.5
Total	3662	14 902 571	24.6	17	3 662 580	0.464	52.9

^a^ This estimate of pregnancy-related mortality is based on the following *International Statistical Classification of Diseases and Related Health Problems, Tenth Revision* codes: A34, O10-O16, O20-O26.7, O26.9-O29.9, O30-O48, O60-O75, O85-O92, O96, O98-O99.7. This excludes pregnancies with abortive outcomes (O00-O07), and non-specific causes of pregnancy-related death (O26.8, O95, O99.8).

**Table 3.** Ratio of Pregnancy-Related Mortality to Abortion-Related Mortality After Removing Nonspecific Causes of Pregnancy-Related Death and COVID-19–Related Deaths, 2018-2021^a^

Year	Pregnancy-related deaths, No.	Births (live births and still births), No.	Pregnancy-related mortality rate, No. per 100 000 births	Abortion-related deaths, No.	Abortions, No.	Abortion-related mortality rate, No. per 100 000 abortions	Ratio
2018	639	3 814 053	16.8	2	885 800	0.226	74.2
2019	750	3 768 908	19.9	4	916 460	0.436	45.6
2020	809	3 634 363	22.3	6	930 160	0.645	34.5
2021	867	3 685 247	23.5	5	930 160	0.538	43.8
Total	3065	14 902 571	20.6	17	3 662 580	0.464	44.3

^a^ This estimate of pregnancy-related mortality is based on the following *International Statistical Classification of Diseases and Related Health Problems, Tenth Revision* codes: A34, O10-O16, O20-O26.7, O26.9-O29.9, O30-O48, O60-O75, O85-O92, O96, O98-O99.7. This excludes pregnancies with abortive outcomes (O00-O07), non-specific causes of pregnancy-related death (O26.8, O95, O99.8), and records where COVID-19 (U07.1) was listed as a multiple cause of death.

**Table 4.** Ratio of Pregnancy-Related Mortality to Abortion-Related Mortality Using Pregnancy Mortality Surveillance System Data to Measure Both Abortion- and Pregnancy-Related Mortality, 2018-2021^a^

Year	Pregnancy-related deaths, No.	Births (live births and still births), No.	Pregnancy-related mortality rate, No. per 100 000 births	Abortion-related deaths, No.	Abortions, No.	Abortion-related mortality rate, No. per 100 000 abortions	Ratio
2018	655	3 814 053	17.2	2	885 800	0.226	76.1
2019	661	3 768 908	17.5	4	916 460	0.436	40.2
2020	904	3 634 363	24.9	6	930 160	0.645	38.6
2021	1222	3 685 247	33.2	5	930 160	0.538	61.7
Total	3442	14 902 571	23.1	17	3 662 580	0.464	49.8

## Discussion

In this cross-sectional study, we found that the ratio of pregnancy- to abortion-related mortality in 2018 to 2021 was between 44.3 and 69.6, at least 3 times higher than the ratio of 14.7 calculated using data from 1998 to 2005.^2^ This change was driven by higher pregnancy-related mortality rates (20.6 to 32.3 vs 10.4) and a lower abortion-related mortality rates (0.46 vs 0.60) relative to the earlier estimate.^2^

Some policy and academic experts have argued that abortion bans themselves will increase pregnancy-related mortality by denying some people abortion care, therefore exposing them to increased health risks from ongoing pregnancy.^26,27^ Therefore, the ratio between pregnancy- and abortion-related mortality may increase in the years after the *Dobbs* decision. At the same time, policy decisions have made measuring the number and causes of pregnancy-related deaths increasingly difficult. Since 2022, 3 states with abortion restrictions have delayed or disbanded their Maternal Mortality Review Committees (MMRCs).^28^ MMRCs provide a more comprehensive review of causes of pregnancy-related deaths than either PMSS or NVSS because they examine a wider record range (including autopsy reports, hospital discharge records, and social service and law enforcement records); however, MMRCs procedures vary by state, limiting the usefulness of their findings for national analysis.^29^ Moreover, the CDC’s most recently reported data on abortion-related mortality are from 2021. As two-thirds of the Division of Reproductive Health, which produces these reports, was eliminated under the recent federal workforce reductions,^30^ it is not clear whether or when additional data will be released or how this reduction will affect PMSS reporting.

### Limitations

This study faced several limitations. First, we could only use 4 years of data (2018-2021). We began the analysis in 2018 because a pregnancy checkbox was not implemented in all states until that year. We ended the analysis in 2021 because that was the most recent year of data on abortion-related mortality released by the CDC. This means that our data do not capture the years after the *Dobbs* decision, which made complete abortion bans legal at the state level. However, as this study captures the pre-*Dobbs* context, our findings can be used for monitoring changes in the pregnancy- to abortion-related mortality ratio going forward. Second, abortion-related mortality is rare in the US (ranging from 2-6 deaths per year during the study period). These low annual numbers mean that a difference of 1 or 2 deaths causes the abortion morality rate to fluctuate widely, leading to high annual variance in the rate. The variance in annual abortion-related deaths, in turn, creates high variance in the annual ratio of pregnancy-related to abortion-related mortality. A moving mean could be used to avoid the sense that the rate was changing so dramatically from year to year; however, the limited number of years available for use in this study prevented us from taking this approach.

An additional limitation of our study is that it is not possible to create an ideal pregnancy-related mortality ratio using available US data. No data source exists in the US that collects a complete count of pregnancies occurring annually in the US.^31^ Therefore, measurement of pregnancy-related mortality in the US is limited to using annual births, rather than annual pregnancies, in the denominator. The ideal numerator for this ratio would exclude maternal deaths among people who did not go on to give birth. This is currently not possible using either of the available data sources capturing annual data on pregnancy-related mortality in the US (NVSS and PMSS). While NVSS does not provide a mortality breakdown by pregnancy outcome, the system does provide *ICD-10* cause-of-death codes which we used to examine pregnancy-related mortality across varied analytic assumptions to account for timing of death during pregnancy, data limitations, and the COVID-19 pandemic. Additionally, there is no criterion standard pregnancy-related mortality data source. Relative to NVSS, pregnancy-related mortality statistics from PMSS data represent deaths in which the pregnancy was confirmed by other sources (eg, pregnancies of >20 weeks via linkage to fetal death or birth records) resulting in lower annual maternal mortality rates.^32^ While this may remove some false positives, PMSS may also fail to capture some true pregnancy-related deaths that cannot be confirmed using available records.

## Conclusions

In this cross-sectional study, we found that the risk of mortality from ongoing pregnancy was far greater than the risk associated with induced abortion in 2018 to 2021. Our estimate was at least 3 times higher than the previous estimate published in 2012,^2^ a change that was likely driven in large part by more complete reporting of maternal deaths. Evidence indicates that the *Dobbs* decision was associated with an increase in births in states with abortion bans, suggesting that bans have forced some people to remain pregnant who would have otherwise sought abortion care.^33,34^ In the post-*Dobbs* environment, such individuals face a substantially greater mortality risk because they remain pregnant despite initially planning for an abortion.

## Notes

Supplement.
Data Sharing Statement
