# Supplementary material for: Pregnancy- and Abortion-Related Mortality in the US, 2018-2021
Source: JAMA Netw Open. 2026 Jan 21;9(1):e2554793. doi: 10.1001/jamanetworkopen.2025.54793 (PMC12824786; doi:10.1001/jamanetworkopen.2025.54793)
Supplement: Supplement. — Data Sharing Statement [file jamanetwopen-e2554793-s001.pdf]

# Data Sharing Statement

Steenland. Pregnancy- and Abortion-Related Mortality in the US, 2018-2021. *JAMA Netw Open*. Published January 21, 2026. doi:10.1001/jamanetworkopen.2025.54793

**Data**
**Data available:** No

**Additional Information**
**Explanation for why data not available:** All of the data included in this study is publicly available. Each data source, and its location, is referenced in the text and reference list.
